# Existential isolation and well-being in justice-involved populations

**DOI:** 10.3389/fpsyg.2022.1092313

**Published:** 2022-12-14

**Authors:** Thomas B. Sease, Cathy R. Cox, Kevin Knight

**Affiliations:** ^1^Institute of Behavioral Research, Texas Christian University, Fort Worth, TX, United States; ^2^Department of Psychology, Texas Christian University, Fort Worth, TX, United States

**Keywords:** existential isolation, justice populations, health, well-being, counseling

## Abstract

Much work in psychology has focused on feelings of social isolation and/or loneliness. Only recently have psychologists begun to explore the concept of existential isolation (EI). EI is the subjective sense that persons are alone in their experience and that others are unable to understand their perspective. EI thus occurs when people feel that they have a unique worldview unshared by others. Measured as either a state or trait, empirical studies have shown EI undermines life meaning and decreases well-being; people scoring high on EI report lower levels of need satisfaction, purpose in life, and meaningfulness and increased death-related concerns. There is also a positive correlation between EI and anxiety, depression, and suicidal ideation. The purpose of this perspective paper is to review literature on EI and discuss its relevance to people who have been involved with the justice system. Given their higher rates of substance use, mental health difficulties, and trauma, this traditionally underserved population is particularly susceptible to compromised well-being. We theorize that EI may impede the impact of therapeutic interventions in justice settings as more isolated individuals may feel disjointed from their counselors and peers, thereby decreasing levels of treatment engagement, participation, satisfaction, and perceived social support. Professionals may be able to mitigate issues related to EI by an enhanced focus on establishing authenticity within the therapist-client relationship (e.g., empathy, perspective taking, compassion), connecting with clients *via* I-sharing [i.e., matching on a shared experience(s)], and/or encouraging active participation in client’s behavioral healthcare needs (e.g., self-reflection).

## Introduction

In the United States, 6.9 million people are on probation, in prison, in jail, or on parole; 9 million persons cycle through local jails; and more than 600,000 people are released from state and federal prisons annually (Assistant Secretary for Planning and Evaluation [ASPE], 2022). With the high number of individuals being released back into the community, pinpointing ways to improve their psychosocial functioning is important toward achieving community reintegration. For instance, more than two-thirds of people are re-arrested within 3 years of their release while half are reincarcerated (Assistant Secretary for Planning and Evaluation [ASPE], 2022). Persons in the justice system are also at greater risk of poverty, death, emotional and physical distress, and have higher suicide rates as compared to the general population (Binswanger et al., 2007; Rosen et al., 2008; Pratt et al., 2010). The current perspective paper will discuss the concept of existential isolation (EI; i.e., feeling alone in one’s experience; Pinel et al., 2017) and how it may function for people belonging to this justice-involved population. This article begins with an overview of EI, a review of current research findings, and how to distinguish it from other forms of isolation (e.g., Yalom, 1980; Helm et al., 2019a). The manuscript then explores the therapeutic importance of EI in individuals in the justice system—a population especially susceptible to existential concerns.

## Existential isolation: Theory and research

Existential isolation occurs when a person feels nobody understands or shares their worldview (Pinel et al., 2017). According to Yalom (1980, p. 355), EI is the “unbridgeable gulf between oneself and any other being.” Although EI is generally approached as a trait-like emotion (e.g., Pinel et al., 2017), one can also have an existentially isolating state (e.g., Helm et al., 2019a). This has led researchers to propose a state-trait model of EI to further understand the antecedents and consequences of each (Helm et al., 2019a). For instance, an acute isolating experience, such as laughing at a movie that no one else finds funny or feeling misunderstood by someone during a conversation, can result in state levels of EI. Negativity from situationally activated EI can motivate persons to reduce the aversive experience(s). For example, experimentally priming EI (vs. loneliness or boredom) leads to a higher accessibility of death-related thoughts (Helm et al., 2019b) and feeling more interpersonally disconnected from others (Pinel et al., 2017). Additionally, although yet to be empirically established, state EI is theorized to be associated with lower self-esteem and perceived meaning in life (Helm et al., 2019a), higher feelings of loneliness and sadness (Helm et al., 2019a), and/or a loss in self-identity as individuals avoid isolation by matching their beliefs with those of others around them (e.g., Swann et al., 2012). Given that the situational experiences of EI should be short-term, the effects of such should also be brief.

If someone is unsuccessful at reducing state levels of EI, or has a repeat number of acute experiences, this may lead to a sustained, trait-like disposition. The personality characteristic of EI is defined by someone feeling that other people, in general, do not understand their subjective experience(s). This may be a consequence of repeat EI inducing events, socialization factors (e.g., avoidant attachment; Helm et al., 2020a), and/or acculturation (Park and Pinel, 2020). Whereas people in a state of EI try to reduce the experience, high scoring existentially isolated persons are more likely to withdraw socially and report greater feelings of hopelessness. Indeed, trait-based EI is negatively related to self-worth, support of communal values (e.g., altruism, trust; Helm et al., 2018), life purpose (Helm et al., 2019a), and “Big 5” personality traits (i.e., conscientiousness, agreeableness, emotional stability, openness to experience, and extroversion; Pinel et al., 2017). Additional findings show that individual differences in EI are positively correlated with anxiety (i.e., generalized, social), self-concealment, stress, and depression (Costello and Long, 2014; Constantino et al., 2019; Helm et al., 2020b). Not only does EI predict higher levels of depression and suicide ideation among college students and Amazon Mechanical Turk (MTurk) samples (Constantino et al., 2019; Helm et al., 2020b), these effects are moderated by dispositional feelings of loneliness; people most susceptible to lower well-being are both lonely and existentially isolated.

Recent evidence also suggests that EI varies demographically. Multiple experiments demonstrate that males report being more existentially isolated than females (Pinel et al., 2017; Helm et al., 2018). This may be a byproduct of socialization and prevailing cultural norms whereby males are discouraged from expressing their emotions and/or indicating a strong need for close others (Helm et al., 2019a). Females, in turn, report lower levels of EI, are more group-focused in their orientation (i.e., equality, loyalty), and are more prosocially motivated (i.e., altruistic, forgiving; Helm et al., 2018). Experimental studies have additionally shown that people belonging to underrepresented communities report higher levels of EI than do persons belonging to majority groups. For example, Pinel et al. (2022) investigated the relationship between non-normative group membership, including race, ethnicity, sexuality, and experiences of EI across three studies. Participants with a non-normative group membership (e.g., gay men and lesbians, Latinas/Latinos) reported higher levels of EI than those with normative group membership (e.g., heterosexuals, non-Latinos/non-Latinas). At a cultural level of analysis, EI is negatively associated with collectivism and identity importance in South Korean participants (Park and Pinel, 2020), while Americans’ higher individualism scores are related to greater feelings of EI (Helm et al., 2019a).

Another goal of EI research is to distinguish the construct from other types of isolation. Yalom (1980), an existential psychotherapist, proposed three forms of separation: (a) intrapersonal, (b) interpersonal, and (c) existential. Intrapersonal isolation refers to aspects of one’s psyche whereby persons are disconnected from themselves in some way (e.g., dissociative disorder, repression). Interpersonal isolation is when someone experiences a lack of social contact or connection with others. This may include, for example, complete separation (e.g., staying at home by oneself for days on end), lacking direct contact (e.g., sitting by oneself in a crowded room), and/or missing meaningful connections with other people (e.g., not establishing deep or long-standing relationships). Regardless of whether interpersonal isolation is self- or other-initiated, people in this state feel emotionally and physically disconnected from others. Finally, no matter how much people may try to bond with friends and family through common interests, shared thoughts and feelings, or similar backgrounds (e.g., upbringing), humans are uniquely alone in their sensory experiences, their interpretation of them, and the extent to which meaning is derived (Pinel et al., 2017). It is this disconnect, according to Yalom (1980) and contemporary researchers (e.g., Pinel et al., 2017; Helm et al., 2019a), that makes EI stand apart from interpersonal isolation in that persons feel chronically and experientially distant from others—a separation beyond meaningful and enduring social relationships.

Research has demonstrated that, although somewhat related, EI and interpersonal isolation are distinct concepts. For instance, with the creation and validation of the Existential Isolation Scale, Pinel et al. (2017) found that trait EI had small but positive correlations with measures of loneliness, alienation, and extraversion. The relationship between variables provided evidence for divergent validity too as EI was unrelated to social desirability and a need to belong; and the magnitude of correlations between variables were much higher for alienation and loneliness compared to feelings of EI. Importantly, other work has demonstrated a direct relationship between EI and impaired physical health (e.g., Costello and Long, 2014), reduced psychological well-being (e.g., anxiety, stress; Constantino et al., 2019; Helm et al., 2020b,2022a), and greater interpersonal dysfunction (e.g., aggression; Pinel et al., 2022), even when controlling for interpersonal isolation.

One limitation of work on EI is that a large proportion of findings have been based on college student samples or participants on cloud-based research platforms, such as MTurk. Although informative, these data do not lend themselves to applied domains because of generalizability concerns. In other words, it could be argued that EI is experienced differently by clinical populations, or that EI affects clinical samples differently. Indeed, theorists have long proposed EI to be a salient psychological concern for clients in psychotherapy (Yalom, 1980; May and Yalom, 1989; Helm et al., 2022a), and empirical studies have shown that people belonging to underrepresented backgrounds are especially vulnerable to feelings of EI (Helm et al., 2022b; Pinel et al., 2022). Despite this, there remains little discussion around the application of EI to clinical samples with more diverse backgrounds.

## Justice-involved populations and existential isolation

Justice-involved populations is a term used to describe people who have previously, or are currently, involved in the justice system. This may include, for example, individuals who are on probation, parole, or serving a sentence in jail or prison. Collectively, justice populations represent an underserved population for behavioral healthcare needs—notably, people involved with the justice system report elevated rates of substance use, mental health difficulties, and trauma histories (Fazel and Seewald, 2012; Fazel et al., 2017; Baranyi et al., 2018). Unfortunately, many persons do not receive services when passing through the justice system because of organizational barriers precluding their delivery (see, e.g., Farabee et al., 1999). Furthermore, the difficulty associated with accessing justice populations in research (e.g., Ferszt and Chambers, 2011; Apa et al., 2012; Charles et al., 2016), in combination with negative attitudes held by the public about justice populations (e.g., Freeman, 2001; Kjelsberg et al., 2007), has likely deterred researchers from investigating the correlates of EI in this at-risk group.

To date, limited work has investigated existential concerns in justice populations. Researchers have explored topics such as concerns about death or the process of finding meaning in life while incarcerated (e.g., Aday, 2006; Aday and Wahidin, 2016; Vanhooren et al., 2016, 2017); however, no work (to our knowledge) has examined the role of EI among people involved with the justice system. It is probable, however, that this population is especially at-risk for experiences of EI. People who are justice involved often experience stigma about having a history of crime, and perceived stigma is associated with diminished psychological well-being (Baffour et al., 2021), willingness to seek treatment for mental health (Clement et al., 2015), and more self-reported interpersonal isolation (Fekete et al., 2018). Conceptually, individuals experiencing stigma related to justice involvement may feel disconnected from their peers and be less likely to engage in behaviors to ameliorate feelings of EI because of concerns about stereotyping, negative judgments, or lack of empathy.

Exacerbating the isolation stemming from stigmatization are the number of people in the justice system with a history of trauma, peer victimization, and/or abuse in childhood. In a study including more than 5,000 persons, 48% of them in prison met the diagnostic criteria for Post-Traumatic Stress Disorder (PTSD) as compared to 4% of individuals in the general population (Briere et al., 2016). Likewise, a meta-analysis reported that 65.7% of people incarcerated in Canada had a history of childhood maltreatment, including physical, emotional, or sexual abuse (Bodkin et al., 2019). Individuals with trauma histories experience difficulties with emotion regulation (Seligowski et al., 2015), are more likely to have anxious or avoidant attachment styles (Woodhouse et al., 2015), and may develop a generalized distrust of others (Hepp et al., 2021). As such, the psychological and emotional difficulties accompanied with a history of trauma may predispose persons to feel more isolated from others without similar experiences, and over time, develop higher levels of trait-based EI that is pervasive across social situations.

Finally, people belonging to racial and ethnic minority groups have been historically overrepresented in justice populations. Although a discussion on the reasons behind this is beyond the scope of this article (see, e.g., Bowman, 2014 for a comprehensive review), a report from the Federal Bureau of Prisons (2022) estimated that 37.5% of individuals in the United States serving time in federal prison were Black, despite only making up 13.4% of the US population. As mentioned, persons who belong to minority, underrepresented, and stigmatized groups report elevated levels of EI as compared to their mainstream societal counterparts (e.g., Pinel et al., 2022). Given that elevated EI is associated with more physical health impairments (e.g., Long et al., 2021) and mental health concerns (e.g., Helm et al., 2020b), it could be that minority people in the justice system are at even greater medical risk as compared to their non-minority counterparts. Although yet to be explored, a study using a MTurk sample found that people scoring high on EI expressed fewer intentions to seek therapy, were less satisfied with mental health treatment, and had lower faith in the expertise of therapists (Constantino et al., 2019).

## Problems and solutions of existential isolation in clinical settings

With the high rates of behavioral health problems in justice populations, residential and outpatient rehabilitation programs aim to provide persons with clinical services to increase psychosocial functioning and reduce individuals’ likelihood of returning to criminal activity. EI, however, may serve as a barrier to providing effective treatment to justice populations as EI is associated with fewer intentions to seek help (Constantino et al., 2019). Namely, trait-based EI may create an inherent disconnect between the therapist and client thereby decreasing attendance, participation, and overall satisfaction with treatment. In group and individual therapy sessions, state-based EI may be precipitated by situational cues of closeness, vulnerability, or perceptions of judgment or misunderstanding from other group members, particularly when individuals view themselves differently from other justice-involved individuals. Persons in treatment settings where state-based EI is evoked may disengage from treatment as an avoidance strategy to protect oneself against the negative feelings associated with being misunderstood. Therapists should therefore consider the use of techniques at treatment onset, such as the use of Motivational Interviewing, to overcome phenomenological isolation and increase clients’ engagement, attendance, and success with treatment. Although our discussion focuses on overcoming EI in clinical settings, we also believe the strategies outlined herein could be used in community settings by people working as social workers, case managers, or medical providers working with this population to provide physical healthcare or social services (e.g., employment, housing).

One strategy that may be useful in overcoming EI in counseling contexts is establishing an authentic therapeutic alliance. The therapeutic alliance (or counselor rapport in some literature) refers to the ongoing working relationship between the client and therapist. Counselor rapport has been associated with more engagement and retention in substance use treatment (Meier et al., 2005), and lower ratings of counselor rapport is associated with less positive treatment outcomes in substance use treatment (i.e., more self-reported substance use) and criminality at a 6-month follow up (Joe et al., 2001). In this way, counselor rapport may serve as an important milestone in therapy that can provide clients with reassurance that the therapeutic setting is a trusting, compassionate, and empathic environment where the client can work on challenges experienced outside the room without a fear of judgment.

Clinicians may rely on processes, such as I-sharing or language matching, to establish an authentic relationship with clients. I-sharing is the sharing of one’s phenomenological experience about said event or situation to achieve a shared reality with another person (Pinel et al., 2010) and may help practitioners relate with clients on a more personal level. I-sharing has been related to positive interpersonal outcomes in experimental studies (e.g., selflessness, agreement with a romantic partner; Huneke and Pinel, 2016; Gehman et al., 2022), and has been theorized to be a mechanism to overcome feelings of EI in clinical settings (Pinel, 2018). Relatedly, language matching may serve as a subtle cue to clients that the therapist is able to understand their experiences. In fact, patient and peer navigators - people with shared experiences that work with people in the justice system overcome challenges with employment, housing, and healthcare needs following a period of justice involvement—have been shown to be an effective strategy at improving client outcomes (Binswanger et al., 2015; Westergaard et al., 2019). The utility of navigators could be in part due to their shared experience and capacity to language match with the client demonstrating a unique understanding of the experience of having been justice-involved.

Another strategy clinicians may use to overcome EI is directly engaging clients in the process of their own therapeutic change. The perception of autonomy, for example, has been theorized to have an important role in intrinsically motivating people for behavioral change (see Ryan and Deci, 2017). Thus, therapists may attempt to provide individuals with a sense of autonomy to overcome feelings of EI and subsequently motivate clients to participate in treatment sessions or activities. Affording autonomy to a client in justice settings may be particularly useful considering many persons may feel a lack of autonomy over other aspects of their life (e.g., sentencing, parole terms). As an example, therapists taking a client-centered approach may ask the client what their personal goals are for treatment, include clients in the planning of therapy sessions, and check in with the client to see how they perceive their progress in treatment (e.g., Johnson and Smalley, 2019). Other subtle ways that therapists may impart clients a sense of autonomy would be to ask for a client’s consent before discussing a difficult topic or warning a client that today’s session will challenging. Through these interactions, therapists may be able to increase clients’ trust in the counselor, the therapeutic process, and their capacity to change behaviors the client deems inconsistent with their goals or values.

## Conclusion

In closing, justice-involved populations represent an at-risk group for EI, which may affect their responsiveness to treatment services designed to improve their overall psychosocial functioning. Existential isolation may impede the therapeutic process as more isolated individuals feel disjointed from their counselors and peers, perceiving decreased levels of social support, and be less likely to engage or participate in treatment services. Professionals may be able to circumvent issues related to EI by establishing authenticity within the counseling relationship (e.g., empathy, perspective taking), connect to clients *via* I-sharing (i.e., matching on shared experiences), and/or encouraging active participation in people’s behavioral healthcare (e.g., self-reflection, granting autonomy). Overall, through a discussion of EI, this paper presents a novel application of EI to justice populations with direct implications for clinical providers to develop remedies for EI in treatment settings.

## Data availability statement

The original contributions presented in this study are included in the article/supplementary material, further inquiries can be directed to the corresponding author.

## Author contributions

TS and CC conceived the original manuscript idea. All authors contributed to the article and approved the submitted version.

## References

[B1] Aday R. H. (2006). Aging prisoners’ concerns toward dying in prison. *OMEGA J. Death Dying* 52 199–216. 10.2190/CHTD-YL7T-R1RR-LHMN 22612255

[B2] Aday R. Wahidin A. (2016). Older prisoners’ experiences of death, dying and grief behind bars. *Howard J. Crime Justice* 55 312–327. 10.1111/hojo.12172

[B3] Apa Z. L. Bai R. Mukherejee D. V. Herzig C. T. Koenigsmann C. Lowy F. D. (2012). Challenges and strategies for research in prisons. *Public Health Nurs.* 29 467–472. 10.1111/j.1525-1446.2012.01027.x 22924569 PMC3694772

[B4] Assistant Secretary for Planning and Evaluation [ASPE] (2022). *Incarceration and reentry.* Washington, DC: U.S. Department of Health and Human Services.

[B5] Baffour F. D. Francis A. P. Chong M. D. Harris N. Baffour P. D. (2021). Perpetrators at first, victims at last: Exploring the consequences of stigmatization on ex-convicts’ mental well-being. *Crim. Justice Rev.* 46 304–325. 10.1177/0734016820960785

[B6] Baranyi G. Cassidy M. Fazel S. Priebe S. Mundt A. P. (2018). Prevalence of posttraumatic stress disorder in prisoners. *Epidemiol. Rev.* 40 134–145. 10.1093/epirev/mxx015 29596582 PMC5982805

[B7] Binswanger I. A. Stern M. F. Deyo R. A. Heagerty P. J. Cheadle A. Elmore J. G. (2007). Release from prison: A high risk factor of death for former inmates. *New Engl. J. Med.* 356 157–165. 10.1056/NEJMsa064115 17215533 PMC2836121

[B8] Binswanger I. A. Whitley E. Haffey P. R. Mueller S. R. Min S. J. (2015). A patient navigation intervention for drug-involved former prison inmates. *Subst. Abus.* 36 34–41. 10.1080/08897077.2014.932320 24960435 PMC4276554

[B9] Bodkin C. Pivnick L. Bondy S. J. Ziegler C. Martin R. E. Jernigan C. (2019). History of childhood abuse in populations incarcerated in Canada: A systematic review and meta-analysis. *Am. J. Public Health* 109 e1–e11. 10.2105/AJPH.2018.304855 30676787 PMC6366501

[B10] Bowman S. W. (ed.) (2014). *Color behind bars: Racism in the US prison system*, Vol. 2. Santa Barbara, CA: Praeger.

[B11] Briere J. Agee E. Dietrich A. (2016). Cumulative trauma and current posttraumatic stress disorder status in general population and inmate samples. *Psychol. Trauma* 8 439–446. 10.1037/tra0000107 26752099

[B12] Charles A. Rid A. Davies H. Draper H. (2016). Prisoners as research participants: Current practice and attitudes in the UK. *J Med Ethics* 42 246–252. 10.1136/medethics-2012-101059 24958334

[B13] Clement S. Schauman O. Graham T. Maggioni F. Evans-Lacko S. Bezborodovs N. (2015). What is the impact of mental health-related stigma on help-seeking? A systematic review of quantitative and qualitative studies. *Psychol. Med.* 45 11–27. 10.1017/S0033291714000129 24569086

[B14] Constantino M. J. Sommer R. K. Goodwin B. J. Coyne A. E. Pinel E. C. (2019). Eistential isolation as a correlate of clinical distress, beliefs about psychotherapy, and experiences with mental health treatment. *J. Psychother. Integr.* 29 389–399. 10.1037/int0000172

[B15] Costello A. E. Long A. E. (2014). “Existential isolation and its psychological and physical health correlates,” in *Poster presentation at the Annual Meeting of the American Psychological Association*, Washington, DC.

[B16] Farabee D. Prendergast M. Cartier J. Wexler H. Knight K. Anglin M. D. (1999). Barriers to implementing effective correctional drug treatment programs. *Prison J.* 79 150–162. 10.1177/0032885599079002002

[B17] Fazel S. Seewald K. (2012). Severe mental illness in 33 588 prisoners worldwide: systematic review and meta-regression analysis. *Brit. J. Psychiat.* 200 364–373. 10.1192/bjp.bp.111.096370 22550330

[B18] Fazel S. Yoon I. A. Hayes A. J. (2017). Substance use disorders in prisoners: an updated systematic review and meta-regression analysis in recently incarcerated men and women. *Addiction* 112 1725–1739. 10.1111/add.13877 28543749 PMC5589068

[B19] Federal Bureau of Prisons (2022). *Inmate statistics.* Available online at: https://www.bop.gov/about/statistics/statistics_inmate_race.jsp (accessed October 30, 2022).

[B20] Fekete E. M. Williams S. L. Skinta M. D. (2018). Internalised HIV-stigma, loneliness, depressive symptoms and sleep quality in people living with HIV. *Psychol. Health* 33 398–415. 10.1080/08870446.2017.1357816 28749185

[B21] Ferszt G. G. Chambers A. (2011). Navigating the challenges of prison research with women. *Nurs. Educ.* 36 256–259. 10.1097/NNE.0b013e3182333f49 22024680

[B22] Freeman R. M. (2001). Here there be monsters: Public perceptions of corrections. *Correct Today* 63 108–111.

[B23] Gehman R. M. Pinel E. C. Johnson L. C. Grover K. W. (2022). Emerging ideas. A ripple effect: Does I-sharing with a stranger promote compromise in cohabiting couples? *Fam. Relat.* 10.1111/fare.12677 PMC10282730 37351021

[B24] Helm P. J. Chau R. F. Greenberg J. (2022a). “Existential isolation: Theory, empirical findings, and clinical considerations,” in *Existential concerns and cognitive-behavioral procedures*, eds Menzies R. G. Menzies R. E. Dingle G. A. (Cham: Springer), 95–113. 10.1007/978-3-031-06932-1_6

[B25] Helm P. J. Jimenez T. Carter S. Arndt J. (2022b). A phenomenological divide: Reference group consequences for existential isolation. *Pers. Soc. Psychol. B*. 1–15. 10.1177/01461672221127799 36214058

[B26] Helm P. J. Greenberg J. Park Y. C. Pinel E. C. (2019a). Feeling alone in your subjectivity: Introducing the state trait existential isolation model (STEIM). *J. Theor. Soc. Psychol.* 3 146–157. 10.1002/jts5.41

[B27] Helm P. J. Lifshin U. Chau R. Greenberg J. (2019b). Existential isolation and death thought accessibility. *J. Res. Pers.* 82:103845. 10.1016/j.jrp.2019.103845

[B28] Helm P. J. Jimenez T. Bultmann M. Lifshin U. Greenberg J. Arndt J. (2020a). Existential isolation, loneliness, and attachment in young adults. *Pers. Indiv. Differ.* 159:109890. 10.1016/j.paid.2020.109890

[B29] Helm P. J. Medrano M. R. Allen J. J. Greenberg J. (2020b). Existential isolation, loneliness, depression, and suicide ideation in young adults. *J. Soc. Clin. Psychol.* 39 641–674. 10.1521/jscp.2020.39.8.641

[B30] Helm P. J. Rothschild L. G. Greenberg J. Croft A. (2018). Explaining sex differences in existential isolation research. *Pers. Indiv. Differ.* 134 283–288. 10.1016/j.paid.2018.06.032

[B31] Hepp J. Schmitz S. E. Urbild J. Zauner K. Niedtfeld I. (2021). Childhood maltreatment is associated with distrust and negatively biased emotion processing. *Bord. Pers. Disord. Emot Dysregul.* 8:5. 10.1186/s40479-020-00143-5 PMC7856450 33536068

[B32] Huneke M. Pinel E. C. (2016). Fostering selflessness through I-sharing. *J. Exp. Soc. Psychol.* 63 10–18. 10.1016/j.jesp.2015.11.003

[B33] Joe G. W. Simpson D. D. Dansereau D. F. Rowan-Szal G. A. (2001). Relationships between counseling rapport and drug abuse treatment outcomes. *Psychiat. Serv.* 52 1223–1229. 10.1176/appi.ps.52.9.1223 11533397

[B34] Johnson L. B. Smalley J. B. (2019). “Engaging the patient: Patient-centered research,” in *Strategies for team science success*, eds Hall K. Vogel A. Croyle R. (Cham: Springer), 135–147.

[B35] Kjelsberg E. Skoglund T. H. Rustad A. B. (2007). Attitudes towards prisoners, as reported by prison inmates, prison employees and college students. *BMC Public Health* 7:71. 10.1186/1471-2458-7-71 PMC1891097 17480213

[B36] Long A. E. Pinel E. C. Park Y. C. Costello A. E. Daily J. R. (2021). *Existential isolation and psychological health in the U.S. and South Korea.* [Manuscript submitted for publication]

[B37] May R. Yalom I. D. (1989). “Existential psychotherapy,” in *Current psychotherapies*, eds Corsini R. J. Wedding D. (Itasca, IL: F E Peacock Publishers), 363–402.

[B38] Meier P. S. Barrowclough C. Donmall M. C. (2005). The role of the therapeutic alliance in the treatment of substance misuse: A critical review of the literature. *Addiction* 100 304–316. 10.1111/j.1360-0443.2004.00935.x 15733244

[B39] Park Y. C. Pinel E. C. (2020). Existential isolation and cultural orientation. *Pers. Indiv. Differ.* 159:109891. 10.1016/j.paid.2020.109891

[B40] Pinel E. C. (2018). Existential isolation and I-sharing: Interpersonal and intergroup implications. *Curr. Opin. Psychol.* 23 84–87. 10.1016/j.copsyc.2018.01.002 29427901

[B41] Pinel E. C. Helm P. J. Yawger G. C. Long A. E. Scharnetzki L. (2022). Feeling out of (existential) place: Existential isolation and nonnormative group membership. *Group Process Interg.* 25 990–1010. 10.1177/1368430221999084

[B42] Pinel E. C. Long A. E. Crimin L. A. (2010). I-sharing and a classic conformity paradigm. *Soc. Cogn.* 28 277–289. 10.1521/soco.2010.28.3.277

[B43] Pinel E. C. Long A. E. Murdoch E. Q. Helm P. (2017). A prisoner of one’s own mind: Identifying and understanding existential isolation. *Pers. Indiv. Differ.* 105 54–63. 10.1016/j.paid.2016.09.024

[B44] Pratt D. Appleby L. Piper M. Webb R. Shaw J. (2010). Suicide in recently released prisoners: A case-control study. *Psychol. Med.* 40 827–835. 10.1017/S0033291709991048 19719900

[B45] Rosen D. L. Schoenbach V. J. Wohl D. A. (2008). All-cause and cause-specific mortality among men released from state prison, 1980-2005. *Am. J. Public Health* 98 2278–2284. 10.2105/AJPH.2007.121855 18923131 PMC2636544

[B46] Ryan R. M. Deci E. L. (2017). *Self-determination theory. Basic psychological needs in motivation, development, and wellness.* New York, NY: The Guilford Press. 10.1521/978.14625/28806

[B47] Seligowski A. V. Lee D. J. Bardeen J. R. Orcutt H. K. (2015). Emotion regulation and posttraumatic stress symptoms: A meta-analysis. *Cogn. Behav. Ther.* 44 87–102. 10.1080/16506073.2014.980753 25421727

[B48] Swann W. B. Jetten J. Gómez Á Whitehouse H. Bastian B. (2012). When group membership gets personal: A theory of identity fusion. *Psychol. Rev.* 119 441–456. 10.1037/a0028589 22642548

[B49] Vanhooren S. Leijssen M. Dezutter J. (2016). Profiles of meaning and search for meaning among prisoners. *J. Posit. Psychol.* 11 622–633. 10.1080/17439760.2015.1137625

[B50] Vanhooren S. Leijssen M. Dezutter J. (2017). Ten prisoners on a search for meaning: A qualitative study of loss and growth during incarceration. *Hum. Psychol.* 45 162–178. 10.1037/hum0000055

[B51] Westergaard R. P. Hochstatter K. R. Andrews P. N. Kahn D. Schumann C. L. Winzenried E. (2019). Effect of patient navigation on transitions of HIV care after release from prison: a retrospective cohort study. *AIDS Behav.* 23 2549–2557. 10.1007/s10461-019-02437-4 30790170 PMC6703963

[B52] Woodhouse S. Ayers S. Field A. P. (2015). The relationship between adult attachment style and post-traumatic stress symptoms: A meta-analysis. *J. Anxiety Disord.* 35 103–117. 10.1016/j.janxdis.2015.07.002 26409250

[B53] Yalom I. D. (1980). *Existential psychotherapy.* New York, NY: Basic Books.

